# Supplementary material for: MAVS-Based Reporter Systems for Real-Time Imaging of EV71 Infection and Antiviral Testing
Source: Viruses. 2023 Apr 26;15(5):1064. doi: 10.3390/v15051064 (PMC10220866; doi:10.3390/v15051064)
Supplement: Supplementary file 1 [file viruses-15-01064-s001.zip › Supplement Information.pdf]

Table S1. Primers used in this study.

Plasmid	Fragment	Forward/Reverse	Sequence
RFP-NLS-MAVSFL	F1	F	ATTTCAGGTGTCGTGAACGCGTGCCACCATGGTGTCTAAGGGCGAAGAGC
		R	TTCAGCAAACGGCATGCTAGCGCCAACTTTTCTTTTCT
	F2	F	GAAAAGAAAAGTTGGCGCTAGCATGCCGTTTGCTGAA
		R	CAGGAATTCTTAATTAAGATATCCTAGTGCAGACGCCGCCGGT
RFP-NLS-MAVS1	F1	F	AGAAAAGTTGGCGCTAGCGACCGTCCCCCAGACCCA
		R	AGGTGAGGGCCTGTGTGTGTCCTGCTCCTGATGCCC
	F2	F	GGGCATCAGGAGCAGGACACACACAGGCCCTCACCT
		R	CAGGAATTCTTAATTAAGATATCCTAGTGCAGACGCCGCCGGT
RFP-NLS-MAVS2	F1	F	AGAAAAGTTGGCGCTAGCGAACTGGGCAGTACCCAC
		R	CCAGGTGAGGGCCTGTGACCCTGTTTACCCTCTGCA
	F2	F	CTGCAGAGGGTAAACAGGGTCACAGGCCCTCACCTGG
		R	CAGGAATTCTTAATTAAGATATCCTAGTGCAGACGCCGCCGGT
RFP-NLS-MAVS3	F1	F	AAAAGAAAAGTTGGCGCTAGCGCAGAGAGTGACCAGGCCG
		R	CCAGGTGAGGGCCTGTGCTTATACTCATTCTCCTCT
	F2	F	AGAGGAGAATGAGTATAAGCACAGGCCCTCACCTGG
		R	CAGGAATTCTTAATTAAGATATCCTAGTGCAGACGCCGCCGGT
RFP-NLS-MAVS4	F1	F	AAAAAGAAAAGAAAAGTTGGCGCTAGCTCCGAGGGCACCTTTG
		R	CAGGAATTCTTAATTAAGATATCCTAGTGCAGACGCCGCCGGT
RFP-NLS-MAVS2-M1	F1	F	ATTTCAGGTGTCGTGAACGCGTGCCACCATGGTGTCTAAGGGCGAAGAGC
		R	GTGAGGCTGGAGGTCGCAGCTGCTGTGT
	F2	F	ACACAGCAGCTGCGACCTCCAGCCTC
		R	CAGGAATTCTTAATTAAGATATCCTAGTGCAGACGCCGCCGGT
RFP-NLS-MAVS2-M2	F1	F	ATTTCAGGTGTCGTGAACGCGTGCCACCATGGTGTCTAAGGGCGAAGAGC
		R	GGATGAGGAGGAGAAGGAGGTGGCAGTAGATACA
	F2	F	TGTATCTACTGCCACCTCCTTCTCCTCCTCATC
		R	CAGGAATTCTTAATTAAGATATCCTAGTGCAGACGCCGCCGGT
RFP-NLS-MAVS2-M3	F1	F	ATTTCAGGTGTCGTGAACGCGTGCCACCATGGTGTCTAAGGGCGAAGAGC
		R	TTTACCCTCTGCAGCCGCTGCAGAGGCCAAGCC
	F2	F	GGCTTGGCCTCTGCAGCGGCTGCAGAGGGTAAA
		R	CAGGAATTCTTAATTAAGATATCCTAGTGCAGACGCCGCCGGT
RFP-NLS-MAVS2-M4	F1	F	AAAGAAAAGTTGGCGCTAGCGAACTGGGCAGTACCCACACAGG
		R	ACGGGATGGTGTGAGGCTGGAGGTCGCAGCTGCTGTG
	F2	F	GCTGCGACCTCCAGCCTCACACCATCCCGTGGGCCTG
		R	GAGGAGGAGAAGGAGGTGGCAGTAGATACAACTGACC
	F3	F	TGTATCTACTGCCACCTCCTTCTCCTCCTCA
		R	ACCCTGTTTACCCTCTGCAGCCGCTGCAGA
	F4	F	GAGGGTAAACAGGGTCACAGGCCCTCACCT
		R	CAGGAATTCTTAATTAAGATATCCTAGTGCAGACGCCGCCGGT
GFP-MAVSFL	F	F	TCTTCCATTTCAGGTGTCGTGAACGCGTGCCACCATGGTGAGCAAGGGCGAG
		R	GTCTTCAGCAAACGGCATGCTAGCCTTGTACAGCTTGTACAGCTCG
GFP-MAVS1	F	F	TCTTCCATTTCAGGTGTCGTGAACGCGTGCCACCATGGTGAGCAAGGGCGAG
		R	GGGTCTGGGGGACGGTCGCTAGCCTTGTACAGCTTGTACAGCTCGTCC
GFP-MAVS2	F	F	TCTTCCATTTCAGGTGTCGTGAACGCGTGCCACCATGGTGAGCAAGGGCGAG
		R	GGGTACTGCCCAGTTCGCTAGCCTTGTACAGCTTGTACAGCTCGTCCAT
GFP-MAVS3	F	F	TCTTCCATTTCAGGTGTCGTGAACGCGTGCCACCATGGTGAGCAAGGGCGAG
		R	CCTGGTCACTCTCTGCGCTAGCCTTGTACAGCTTGTACAGCTCGTCCA
GFP-MAVS4	F	F	TCTTCCATTTCAGGTGTCGTGAACGCGTGCCACCATGGTGAGCAAGGGCGAG
		R	CAAAGGTGCCCTCGGAGCTAGCCTTGTACAGCTTGTACAGCTCGTCCA
GFP-MAVS2-M1	F	F	TCTTCCATTTCAGGTGTCGTGAACGCGTGCCACCATGGTGAGCAAGGGCGAG
		R	GGTACTGCCCAGTTCGCTAGCCTTGTACAGCTTGTACAGCTCGTCCAT
GFP-MAVS-M2	F	F	TCTTCCATTTCAGGTGTCGTGAACGCGTGCCACCATGGTGAGCAAGGGCGAG
		R	GGTACTGCCCAGTTCGCTAGCCTTGTACAGCTTGTACAGCTCGTCCAT
GFP-MAVS-M3	F	F	TCTTCCATTTCAGGTGTCGTGAACGCGTGCCACCATGGTGAGCAAGGGCGAG
		R	GGTACTGCCCAGTTCGCTAGCCTTGTACAGCTTGTACAGCTCGTCCAT
GFP-MAVS-M4	F	F	TCTTCCATTTCAGGTGTCGTGAACGCGTGCCACCATGGTGAGCAAGGGCGAG
		R	GGTACTGCCCAGTTCGCTAGCCTTGTACAGCTTGTACAGCTCGTCCAT

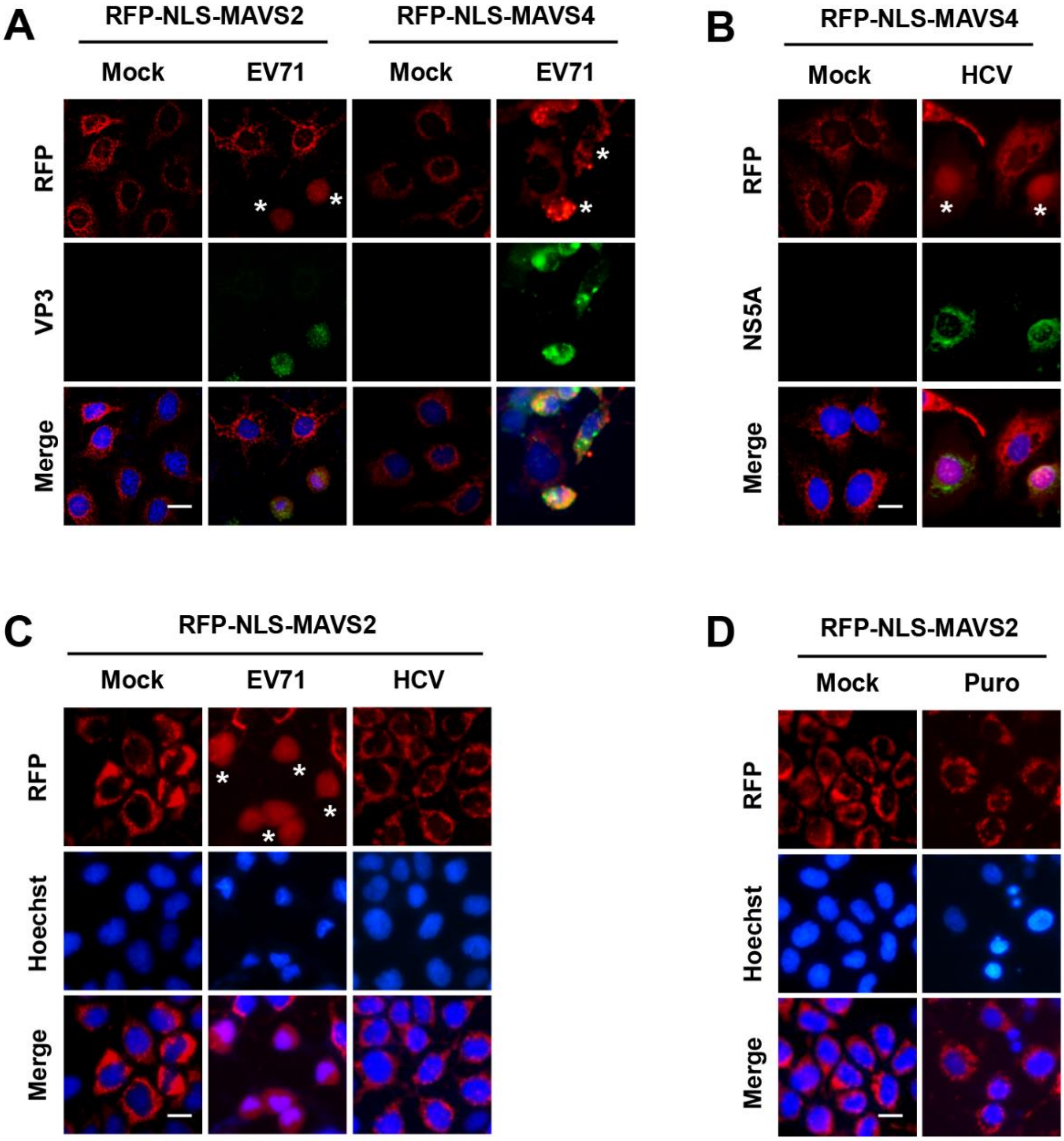

Figure S1. Validation of MAVS-based reporter systems for EV71 infection. (A) Huh 7 cells stably expressing RFP-NLS-MAVS2 or RFP-NLS-MAVS4 were infected with EV71 and immunostained with VP3 antibody followed by fluorescence observation at 20 h post-infection. Nuclei were stained with Hoechst (blue). Asterisks indicate infected cells. Bar, 20 µm.(B) Huh 7 cells stably expressing RFP-NLS-MAVS4 were infected with HCV and immunostained with NS5A antibody followed by fluorescence observation at 72 h post-infection. Nuclei were stained with Hoechst (blue). Asterisks indicate infected cells. Bar, 20 µm. (C) Huh 7 cells stably expressing RFP-NLS-MAVS2 were either mock infected (left), infected with EV71 (middle) or HCV(right) and fluorescence was monitored at 20 h.p.i. (EV71) or 72 h.p.i.(HCV). Nuclei were stained with Hoechst (blue). Asterisks indicate infected cells. Bar, 20 µm. (D) Huh 7 cells stably expressing RFP-NLS-MAVS2 were mock treated or treated with 1 mg/ml puromycin for 24 h and fluorescence was monitored. Asterisks indicate infected cells. Bar, 20 µm.

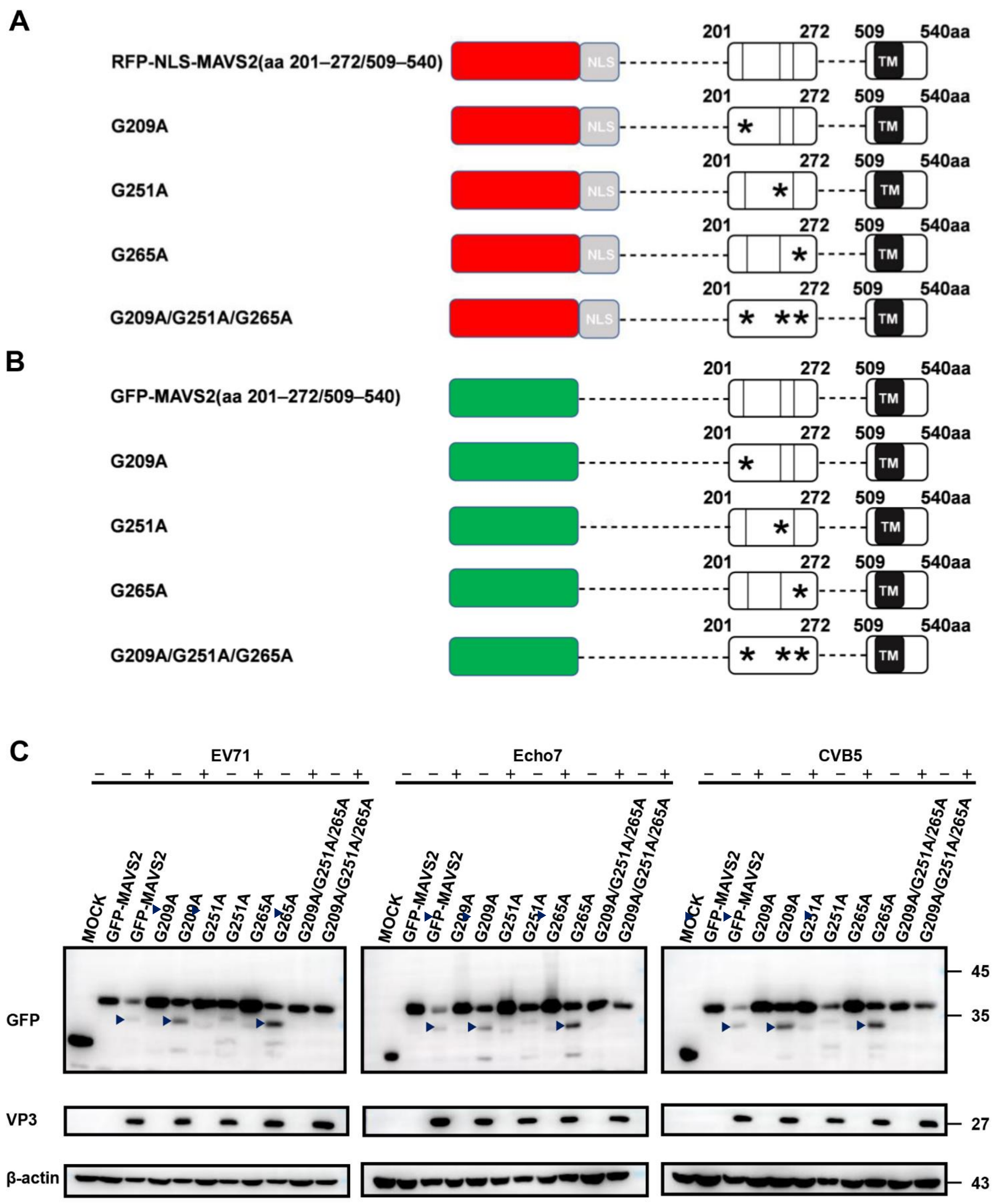

Figure S2. EV71, E7 and CVB5 cleave GFP-MAVS reporter at same amino acid sites. (**A**)Schematic diagram of RFP-NLS-MAVS reporter mutants. Asterisks indicate sites of mutation. (B) Schematic diagram of GFP-MAVS reporter mutants. Asterisks indicate sites of mutation. (C) Huh 7 cells stably expressing GFP-MAVS2 or indicated mutant were infected with EV71, Echo7 or CVB5 and immunoblotted with GFP or VP3 antibody. Arrowheads indicate cleaved MAVS. β-actin was shown as loading control.

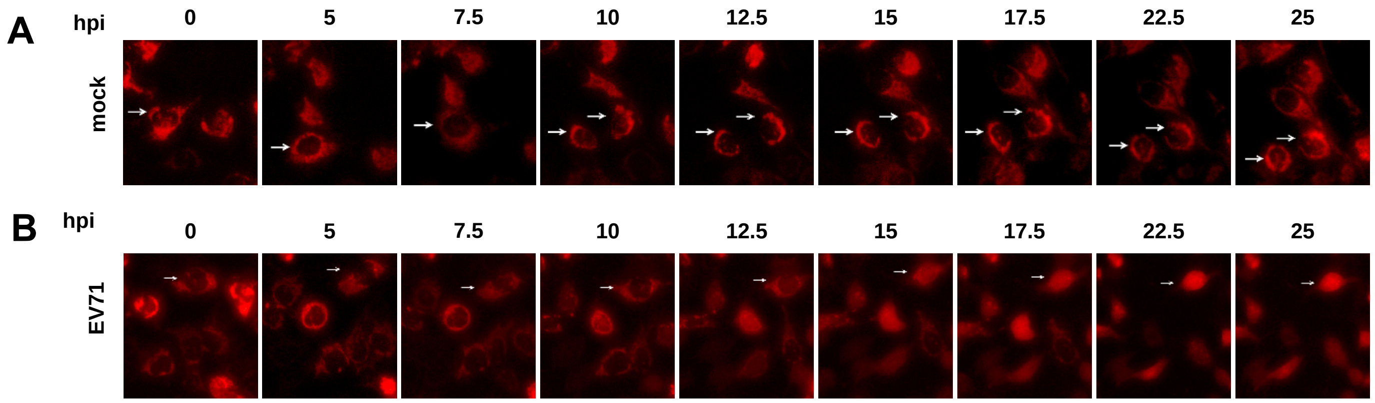

Figure S3. Time-lapse live-cell imaging of EV71 infection. (**A**) Huh 7 cells stably expressing RFP-NLS-MAVS2 were mock infected and fluorescent images were taken every 2.5 h. Arrow indicates one mock infected cell, which divided into two cells around 10 hpi. (**B**) Huh 7 cells stably expressing RFP-NLS-MAVS2 were infected with EV71 (MO = 5) and fluorescent images were taken every 2.5 h. Arrow indicates one EV71 infected cell.

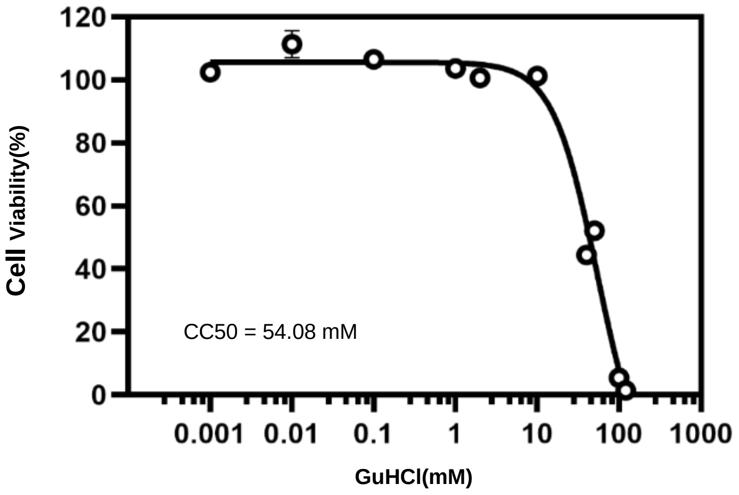

Figure S4. Cytotoxicity of GuHCl on tested cells. Huh 7 cells stably expressing RFP-NLS-MAVS2 were treated with indicated concentration of GuHCl for 24 h and cell viability was determined by CellTiter-Glo® Luminescent Cell Viability Assay (Promega Corporation).
